# Shared and Unique Proteins in Human, Mouse and Rat Saliva Proteomes: Footprints of Functional Adaptation

**DOI:** 10.3390/proteomes1030275

**Published:** 2013-12-16

**Authors:** Robert C. Karn, Amanda G. Chung, Christina M. Laukaitis

**Affiliations:** College of Medicine, University of Arizona, Tucson, AZ 85724, USA; E-Mails: achung@email.arizona.edu (A.G.C.); cmlaukai@email.arizona.edu (C.M.L.)

**Keywords:** human, mouse, rat, saliva, proteome, evolution, adaptation

## Abstract

The overall goal of our study was to compare the proteins found in the saliva proteomes of three mammals: human, mouse and rat. Our first objective was to compare two human proteomes with very different analysis depths. The 89 shared proteins in this comparison apparently represent a core of highly-expressed human salivary proteins. Of the proteins unique to each proteome, one-half to 2/3 lack signal peptides and probably are contaminants instead of less highly-represented salivary proteins. We recently published the first rodent saliva proteomes with saliva collected from the genome mouse (C57BL/6) and the genome rat (BN/SsNHsd/Mcwi). Our second objective was to compare the proteins in the human proteome with those we identified in the genome mouse and rat to determine those common to all three mammals, as well as the specialized rodent subset. We also identified proteins unique to each of the three mammals, because differences in the secreted protein constitutions can provide clues to differences in the evolutionary adaptation of the secretions in the three different mammals.

## 1. Introduction

The advent of genomic and proteomic sciences has provided a flood of new information about genes expressed to produce the array of proteins characteristic of a particular tissue. Determining which genes are expressed in a particular type of cell and/or in the fluid it secretes can be done by assaying either RNA transcripts, translated protein products or, ideally, both. Mammals, including primates and rodents, produce and secrete proteins into saliva from three major salivary glands: the parotid, sublingual and submandibular glands, as well as other minor sources (e.g., tongue). 

Salivary glands produce the proteins necessary to initiate digestion, to lubricate the hard and soft tissues of the mouth and to protect against infection. Primary salivary gland malfunction can occur due to viral or bacterial infection, autoimmune disease (e.g., Sjögren’s syndrome [1]), calcium stone formation, which blocks secretion, or tumor development and/or invasion. Medications and radiation treatment can also inhibit salivary gland function. A decrease in saliva production leads to the breakdown of teeth and the other oral cavity structures, thus much attention is focused on maintaining appropriate salivary gland function. 

We previously obtained saliva proteomes of the genome mouse (C57BL/6) and the genome rat (BN/SsNHsd/Mcwi) using multidimensional protein identification technology (MUDPIT) for the purpose of studying rapidly evolving proteins and their genes [2]. That publication focused on the independent expansions of the mouse and rat kallikrein subfamilies expressed in saliva and how selection influenced their evolution. 

The overall goal of the project we report here was to compare the proteins found in the saliva proteomes of three mammals, human, mouse and rat, in order to identify proteins shared and unique to one or more taxa. We selected two different human saliva proteomes to compare and contrast with our rodent saliva proteomes [2]. One human saliva proteome [3] was produced from whole saliva and analyzed at a depth similar to the rat and mouse proteomes we produced, while the second [4] reported a far more extensive human saliva proteome from salivary gland duct secretions collected by three different groups participating in a consortium. Because these two human proteomes differ both in collection and analysis techniques, our first objective was to compare the identifications made by the two studies. Our questions are:

1. Which proteins are shared between the two human saliva proteomes and which are not?
2. Does a deeper proteome necessarily improve the protein representation of salivary gland secretions?
3. Does using saliva collected from individual salivary gland ducts, rather than whole saliva, improve the representation of salivary gland secretions in the final analysis?

The major advantage of proteomes is that proteins identified at a high probability from two or more high quality peptides can be confidently believed to be present in the protein mixture analyzed. However, in secretions, such as saliva or tears, one cannot conclude that every identified protein was secreted by the gland(s) producing that fluid. Proteins found in saliva are primarily secreted by salivary glands, but can also result from contamination from other sources (e.g., tracheal, naso-pharyngeal) or from cellular breakdown. We used the presence of a signal peptide as a surrogate for extracellular secretion [5] in order to eliminate from further consideration the contaminating proteins most likely produced by cellular breakdown. 

The mouse and rat are widely used as experimental organisms in studies of human pathological conditions, and so, it is important to understand the ways in which their physiologies are comparable to human physiology and the ways in which they are not. Moreover, differences in the secreted salivary proteins can provide clues to differences in the evolutionary adaptation of the secretions in the three different mammals. Thus, our second objective was to determine which salivary proteins are shared among the three mammal proteomes and which are unique to one of them or shared by only two of them. For this objective, our questions were:

1. What proteins are shared by the human, mouse and rat saliva proteomes, and which are shared by two of the three proteomes?
2. Are the proteins shared between two or all three mammal proteomes encoded by genes with known evolutionary relationships, that is to say that they are orthologous or paralogous; or is their apparent similarity an accident of naming that does not represent a true evolutionary relationship?
3. What proteins are unique to the saliva proteomes of each of the three mammals?

It was our ultimate goal to determine whether the proteins that appeared to be similar in two or more mammal saliva proteomes actually shared an evolutionary history, *i.e*., they were orthologous/ paralogous, or whether the similarity was superficial and they do not share an evolutionary history. Superficial similarities can arise when characteristics that may have occurred as the result of convergent evolution (e.g., a high representation of an amino acid, such as proline) result in similar naming, but where a shared evolutionary history is lacking in the two taxa under scrutiny. In discussing potentially shared evolutionary histories, we tried to take into consideration the similarities and differences in rodent and human nutritional physiology and behavior.

## 2. Experimental

### 2.1. Protein Identification from Proteomic Data

The materials and LC-MS/MS methods were reported previously for the human [3,4], mouse (C57BL/6) and rat (BN/SsNHsd/Mcwi) [2] saliva proteomes. The information on rat *Klk1* gene subfamily expression in the Sprague-Dawley strain can also be found in [2]. 

**Figure 1:**
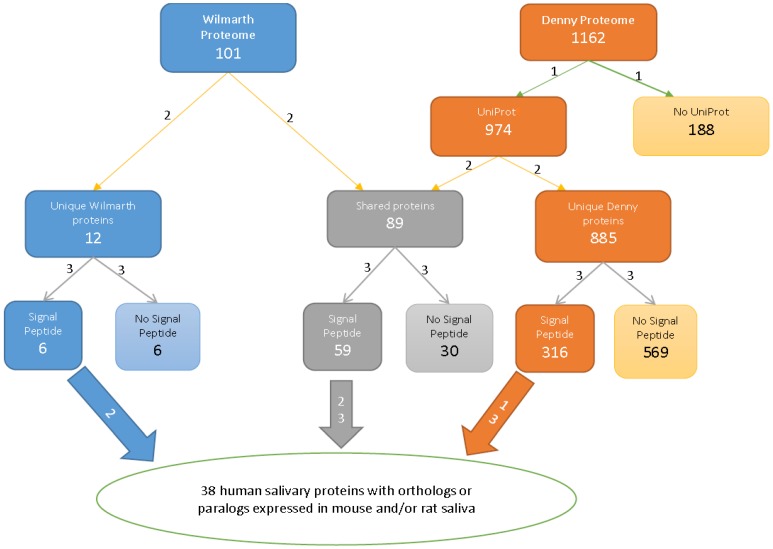
Flow chart for comparing the two human proteomes (Steps 1, 2 and 3) and the human with rodent saliva proteomes. Step 1: the IPI accession numbers of the proteome [4] were converted to UniProt accession numbers; Step 2: proteins in the two proteomes were sorted by their UniProt numbers; Step 3: proteins were grouped by signal peptide status.

The spectra from the two human studies were identified by searching against two different databases, the human-only entries in Swiss-Prot (Swiss-Prot, Release 42.0, October 2003) [3] and the European Bioinformatics Institute (EBI) human International Protein Index (IPI) database (version 3.01; release date November 1, 2004) [4]. To compare these identifications, we first converted the two sets of data to the UniProt format, and this was especially important in view of the deactivation of the IPI database. We used the UniProt ID Mapping function to batch convert IPI numbers [6]. Some IPI numbers could not be converted to UniProt in that way; thus, we used the NCBI protein search function to convert the remaining IPI numbers. One hundred and eighty-eight proteins from [4] were not successfully converted from IPI to UniProt Accession numbers, and these were eliminated from further analysis. Furthermore, some proteins have several IPI numbers that convert to the same UniProt number, and there are also proteins with one IPI number that correspond to multiple UniProt numbers. In those cases, we evaluated each protein number and retained only the validated or most recently reviewed UniProt number. See Figure 1 for a summary of this and downstream processes. 

### 2.2. Sorting Shared and Non-Shared Human Salivary Proteins

Microsoft Access was used to compare the proteins identified in human proteomes [3,4] by designing queries to search for shared UniProt Accession numbers in both proteomes and to search for UniProt numbers unique to each proteome (Figure 1). To identify unique proteins in [4], the UniProt Accession numbers were searched against those found in [3] using “Is Null” criteria. This query was rerun comparing the [3] proteome against the [4] proteome to produce proteins unique to [3].

### 2.3. Identifying Secreted and Non-Secreted Proteins in the Saliva Proteomes

SignalP [7,8] was used to predict the presence or absence of a signal-peptide cleavage site for each protein to help determine whether or not that protein will be processed for secretion (Figure 1). Proteins with a D score greater than 0.45 were predicted to have a signal peptide and signal-peptide cleavage site, designating them as putative secreted proteins. Proteins with a D score below 0.45 were categorized as lacking a signal peptide. 

### 2.4. Identifying Similar Proteins

We grouped the shared human proteins with the most similar rodent proteins by UniProt ID and then tested for the orthology and paralogy of their genes. Orthology between human, mouse and rat were computed using the “orthology” feature on [9], which identifies the best Basic Local Alignment Search Tool for Protein sequences (BLASTP) match and filters out non-syntenic hits [10]. For unclear protein identities, the Genome Browser Convert utility was used to locate the position of a gene in the genome assembly of other species [10]. During the conversion process, portions of the genome in the coordinate range of the original assembly were aligned to the new assembly, while preserving their order and orientation. We double-checked all proteins found only in two of three taxa against the other taxon by identifying the ortholog’s UniProt number with BLASTP and manually searching the appropriate proteome for that protein.

## 3. Results and Discussion

### 3.1. Comparing and Contrasting the Proteins Identified in Two Human Saliva Proteomes

We chose two human saliva proteomes of very different depths to compare and contrast. One study collected whole saliva from a single adult male and separated peptides with two-dimensional chromatography linked to mass spectrometry [3]. The second study was far more extensive, involving three different institutions in a consortium that produced a deeper proteome [4]. In that study, salivas were collected from subjects of both sexes using collection devices designed for each duct. The peptides were separated by a number of different methods before LC-MS/MS analysis of the peptide mixtures was performed. We wished to determine how the results from these two very different human saliva proteome studies compared and contrasted. 

### 3.2. Which Proteins Are Shared between the Two Human Saliva Proteomes and Which Are Not?

Questions 1 and 2 that we posed for the first objective in the Introduction concerned: (1) which proteins are shared between the two human saliva proteomes and which are not; and (2) does a deeper proteome necessarily improve the protein representation of salivary gland secretions? Nearly all of the proteins identified in the shallower proteome (89/101; 88%) were also found by the consortium project (Supplemental File 1; SF1). Figure 1 shows the sorting flow chart. Subsequent SignalP analysis showed that 66% of the shared proteins (59/89) have signal peptides and 34% (30/89) do not. Nearly 2/3 of the proteins uniquely identified by the consortium [4] (569/885; SF2) lack signal peptides, as do 6/12 (50%) of proteins unique to [3] (SF3). We interpret these findings to mean that the shared proteins in this comparison represent a core of highly-expressed human salivary proteins, while those unique to a proteome are at least as likely [3] to twice as likely [4] to be contamination from intracellular and other sources. It probably should be expected that a deeper proteome may reveal less highly-represented proteins, but at the expense of detecting more contaminating proteins. 

In Question 3 of the first objective, we asked whether using saliva collected from individual salivary gland ducts, rather than whole saliva, improved the representation of salivary gland secretions in the final analysis. Given that both human saliva proteomes agreed on most of the proteins identified in the shallower one and that 2/3 of the residual proteins in the deeper proteome lack signal peptides and are likely to be contaminants, we cannot conclude that one collection method was clearly superior to the other. It is probably safer to conclude that the different depths of analysis were more important than the sample collection methods. 

### 3.3. Proteins Shared by Mouse, Rat and Human Saliva Proteomes

Our second objective was to compare the salivary proteins from the proteomes of the three mammals, human, mouse and rat, to determine the subset shared by all three mammals’ saliva, those shared by only two of the three (Table 1, Table 2) and those that are unique to each of the three mammals’ saliva. Genes that are derived by speciation have been defined as orthologs and clearly share a common descent, whereas genes that evolved through duplication are called paralogs ([11,12] and reviewed in [13]). While it is clear that paralogs share an evolutionary history, they lack the direct 1:1 relationship of orthologs and may have different origins in different species. The third possibility is that evolutionarily unrelated proteins may share a common name. This is the null hypothesis against which we are testing potentially-related proteins. 

To begin the comparison, we separated the human saliva proteome into those proteins shared between the two studies [3,4] and those unique to just one. We first grouped the shared human proteins with the most similar rodent proteins by UniProt ID and then tested for the orthology and paralogy of their genes. The residual proteins in the rodent proteomes were then compared to the unique proteins of [3] and [4] and their genes tested for orthology and paralogy. Those proteins left unmatched by these tests were considered to be the sets unique to each of the three mammals. 

Some proteins, such as carbonic anhydrase, kallikrein-1 and nucleobindin, are clearly orthologs in all three mammals (Table 1). In other instances, such as alpha-amylase, two of the three mammals (mouse and rat) have orthologous genes, while the human gene is paralogous. Nonetheless, they clearly share evolutionary histories, as indicated by the fact that they are all located in chromosomal regions that are homologous in the three taxa. Other proteins are structurally related, but non-orthologous. For example, five human proline-rich proteins (PRPs) shared chromosomal region homology with two mice and five rat PRPs, while three human proline-rich proteins, including statherin, have no corresponding proteins in the rodents. 

This part of the study allows us to comment further on the effect of the proteome depth of protein detection. Figure 1 shows that the number of proteins in the pool shared by the human saliva proteome with one or the other rodent proteomes was augmented more than 50% from the [4]-unique collection, but only 10% from the [3]-unique collection. While this supports the idea that a deeper proteome provides an advantage over a shallower one, we also note that a very large number of the residual proteins in the [4]-unique pool appear to be contaminants, as shown by our SignalP analysis. Of additional concern is that the shallower of the two human proteomes found all five members of the Ig secretory complex [3], while the deeper proteome missed the Ig lambda light-chain (Q6GMV7) and Ig alpha-2 chain c region (P01877). Thus, a deeper proteome clearly confers an overall advantage in protein representation, but this may not be true for all proteins. 

**Table 1:** Rodent salivary protein matches with human (orthologous/paralogous genes).

Protein (common name)	UniProt (paralog)	Gene	Human ortholog?	Mouse ortholog?	Rat ortholog?	Criteria
Alpha 2 macroglobulin	Hs P01023	A2MG	--------	N/A	(paralog)	2
	Hs A8K2U0	A2ML1	--------	N/A	(paralog)	2
	Rn D3ZS19	BC048546	(paralog)	N/A	--------	2
Amylase, salivary	Hs P04745	AMY1	--------	(paralog)	(paralog)	1, 2
	Mm P00687	Amy1	(paralog)	--------	Amy1a	1, 2
	Rn Q99N59	Amy1a	(paralog)	Amy1	--------	1, 2
Amylase, pancreatic	Hs P04746	AMY2A	--------	(paralog)	(paralog)	1,2
	Mm P00688	Amy2a2	(paralog)	--------	Amy2	1,2
	Rn P00689	Amy2	(paralog)	Amy2a2	--------	1,2
Angiotensin trypsin-1	Hs P01019	AGT	--------	Agt	N/A	2
	Mm P11859	Agt	AGT	--------	N/A	1,2
BPI fold-containing family A member 2	Hs Q96DR5	BPIFA2	--------	Bpifa2	Bpifa2 (aka Psp)	1
	Mm P07743	Bpifa2	BPIFA2	--------	Bpifa2 (aka Psp)	1
	Rn Q63471	Bpifa2 (aka Psp)	BPIFA2	Bpifa2	--------	1
	Rn Q63550	Bpifa2f	(paralog)	N/A	--------	2
BPI fold containing family B member 1	Hs Q8TDL5	BPIB1	--------	Bpifb1	Bpifb2	1
	Mm Q61114	Bpifb1	BPIP1	--------	Bpifb3	1
	Rn A0JPN3	Bpifb1	BPIP2	Bpifb1	--------	1
Carbonic anhydrase	Hs P23280	CA6	--------	Ca6	Car6	1
	Mm P18761	Ca6	CA6	--------	Car6	1
	Rn F1LQ08	Car6	CA6	Ca6	--------	1
Chromosome 6 (Hs) open reading frame 58	Hs Q6P5S2	C6orf58	--------	2310057J18Rik	(paralog)	1, 2
	Mm Q8C6C9	2310057J18Rik	C6orf58	--------	(paralog)	1, 2
Cystatins	Hs P28325	CST5	--------	(paralog)	(paralog)	1, 2
	Hs P01036	CST4	--------	Cst10	Cst5	1, 2
	Hs P01037	CST1	--------	(paralog)	(paralog)	1, 2
	Hs P01034	CST3	--------	(paralog)	(paralog)	1, 2
	Hs P09228	CST2	--------	(paralog)	(paralog)	1, 2
	Mm Q9JM84	Cst10	Cst4	--------	Cst5	1
	Rn D4AAU9	Cst5	Cst4	Cst10	--------	2
Cysteine-rich protein	Hs P54108	CRISP3	--------	(paralog)	(paralog)	1, 2
	Mm Q03401	Crisp1	(paralog)	--------	Crisp1	1, 2
	Rn P12020	Crisp1	(paralog)	Crisp1	--------	1, 2
(pro)Epidermal growth factor	Hs P01133	EGF	--------	Egf	N/A	2
	Mm P01132	Egf	--------	--------	N/A	2
Gamma-glutamyl hydrolase	Hs Q92820	GGH	--------	Ggh	N/A	1
	Mm Q9Z0L8	Ggh	GGH	--------	N/A	1
Granulin	Hs P28799	GRN	--------	Grn	N/A	1
	Mm P28798	Grn	GRN	---------	N/A	1
Kallikrein 1	Hs P06870	KLK1	--------	Klk1	Klk1	1
	Mm P15947	Klk1	KLK1	--------	Klk1	1
	Rn P00758	Klk1	KLK1	Klk1	--------	1
Lactoperoxidase	Hs P22079	LPO	--------	Lpo	Lpo	1, 2
	Mm Q5SW46	Lpo	LPO	--------	Lpo	1, 2
	Rn D4A400	Lpo	LPO	Lpo	--------	1, 2
Lipocalin 1	Hs P31025	LCN1	--------	N/A	(paralog)	- - - - - -
	Rn P20289	Vegp1	(paralog)	N/A	--------	- - - - - -
Mannosidase, alpha	Hs O00754	MA2B1	Ma2B1	--------	N/A	1
	Mm O09159	Ma2B1	--------	MA2B1	N/A	1
Nucleobindin	Hs P80303	NUCB2	--------	Nucb2	Nucb2	1, 2
	Mm P81117	Nucb2	--------	--------	Nucb2	1, 2
	Rn Q9JI85	NUCB2	(paralog)	Nucb2	--------	1, 2
Polymeric immunoglobulin receptor	Hs P01833	PIGR	--------	Pigr	Pigr	1
	Mm O70570	Pigr	PIGR	--------		1
	Rn P15083	Pigr	PIGR	Pigr	--------	1
Prolactin-inducible protein homolog	Hs P12273	PIP	--------	Pip	Pip	1
	Mm P02816	Pip	PIP	--------	Pip	1
	Rn O70417	Pip	PIP	Pip	--------	1
Proline-rich proteins	Hs P02810	PRH1-PRR4	--------	(paralog)	(paralog)	1, 2
	Hs P04280	PRB1	--------	(paralog)	(paralog)	2
	Hs P02812	PRB2	--------	(paralog)	(paralog)	2
	Hs P10163	PRB4	--------	(paralog)	(paralog)	2
	Hs Q04118	PRB3	--------	Prp2	(paralog)	2
	Mm Q91X93	Prb1	(paralog)	--------	(paralog)	2
	Mm Q58E44	Prpmp5	(paralog)	--------	(paralog)	2
	Rn P10165	Prb1	(paralog)	(paralog)	--------	2
	Rn Q04154	Prp2	(paralog)	(paralog)	--------	2
	Rn Q04105	Prp15	(paralog)	(paralog)	--------	2
	Rn P10164	LOC100362849	(paralog)	(paralog)	--------	2
	Rn Q07610	Prpg1	(paralog)	(paralog)	--------	2
Protease, serine, 1 (trypsin 1)	Hs P07477	PRSS1	--------	--------	Prss1	2
	Rn P00762	Prss1	PRSS1	--------	--------	2
RNase1	Hs P07998	RNASE1	--------	N/A	(paralog)	2
	Rn P00684	Rnase1	(paralog)	N/A	--------	2
Serum albumin	Hs P02767	ALB	--------	Alb	Alb	1
	Mm P07724	Alb	ALB	--------	Alb	1
	Rn P02770	Alb	ALB	Alb	--------	1
Submaxillary gland androgen regulated protein 3A	Hs Q99954	SMR3A	--------	Smr3a	(paralog)	2
	Mm Q61900	Smr3a	SMR3A	--------	(paralog)	2
	Rn P13432	Smr3a/Vcsa1	(paralog)	--------	--------	2
Transcobalamin-2	Hs P20062	TCN2	--------	Tcn2	N/A	1
	Mm O88968	Tcn2	TCN2	--------	N/A	1
Transferrin	Hs P02787	TF	--------	N/A	(paralog)	2
	Rn Q7TP24	Ba1-667	(paralog)	N/A	--------	2
WFDC	Hs Q14508	WFDC2	--------	(paralog)	N/A	1
	Mm Q9JHY3	Wfdc12	(paralog)	--------	N/A	1

**Table 2:** Mouse-rat salivary protein matches (orthologous/paralogous genes).

Protein (common name)	UniProt (paralog)	Gene	Mm ortholog?	Rn ortholog?	Criteria
Chitinase	Mm Q91XA9	Chia	--------	Chia	1
	Mm Q91Z98	Ch3L4	--------	(paralog)	2
	Rn Q6RY07	Chia	Chia	--------	1
Common salivary protein	Mm Q99JV1	Dcpp1	--------	LOC171161	2
	Mm E9PYC2	Dcpp2	--------	LOC171161	2
	Mm L7N259	Dcpp3	--------	LOC171161	2
	Rn Q63015	LOC171161	Dcpp1Dcpp2	- - - - - -	2
DNAS1	Mm P49183	Dnase1	--------	Dnase1	1
	Rn P21704	Dnase1	Dnase1	--------	1
OBP1f-like	Mm Q9D3N5	5430402E10Rik	--------	Obp1F	2
	Rn Q9QYU9	Obp1F	5430402E10Rik	--------	2
Ovostatin	Mm QUU35	Ovos	(paralog)	--------	
	Rn D3Z9M3	LOC362451	--------	(paralog)	
Proline rich, lacrimal 1	Mm E9PYQ4	Prol1	--------	Prol1	1
	Rn Q62605	Prol1	Prol1	--------	1
Submandibular gland protein	Mm Q6JHY2	Smgc	--------	Smgc	2
	Rn Q6JHY3	Smgc	Smgc	--------	2
1 UCSC BLASTP					
2 UCSC Convert tool					

### 3.4. Proteins Unique to Rodent Saliva

Clearly, the three mammals share a core of proteins that play important roles in the early stages of digestion, in protecting and lubricating hard and soft surfaces and in immunological protection and maintenance of the oral cavity generally. Given the many decades of research on individual proteins playing these roles, this is hardly surprising. Perhaps more intriguing are the proteins shared by mouse and rat, but absent from human saliva, especially since the mouse and rat are widely used as experimental organisms in studies of human pathological conditions, and rodent-specific proteins may limit the applicability of these models. The rodent-shared protein group (Table 2) is 25% as large (seven) as the core shared between human and one or both rodents (29; Table 1). Four of the seven rodent-unique proteins are clearly orthologous, while half of the proteins shared between humans and rodents include complex paralog/ortholog sets, reflecting more complex evolutionary histories. 

The mouse and rat secrete chitinase, common salivary protein, deoxyribonuclease, odorant binding protein, ovostatin, proline-rich lacrimal 1 protein and submandibular gland protein into their saliva that humans do not. Other studies have shown that both rodents are capable of expressing an impressive array of kallikreins from subfamilies that are unique to each genome [14] (see below). 

These important differences in secreted salivary proteins may provide clues to differences in the evolutionary adaptation of the secretions in the three different mammals. For example, it is possible that chitinase and deoxyribonuclease in rodent saliva provide the potential for digesting food sources more available to rodents than to humans. We also note that some of the proteins unique to rodent saliva proteomes may play a primary or secondary role in grooming and pelage maintenance. Humans are one of the few mammals without a pelage of fur or wool covering nearly the entire body, and thus, the potential roles of proteins involved in grooming and pelage maintenance are not included in most human-centric discussions of saliva constitution. For example, we have previously shown that mice coat their pelts with salivary androgen-binding protein (ABP; [15]), and we suggested that this was a means of advertising the subspecies of the animal, since ABP has been implicated in mediating subspecies identification [15,16,17,18]. A general role in coating surfaces was later proposed for secretoglobins, such as ABP, by Dominguez [19] following the first report of substantial identities among rabbit uteroglobin, cat Fel dI and mouse ABP by Karn [20]. One can envision that a surface coating might include a chitinase that could defend against ectoparasites by attacking their exoskeletons. 

The presence of the unique array of salivary kallikreins in rodent saliva is a knotty problem, given that, at least in mouse saliva, they show extensive sex-limited expression. Rodent species, including the house mouse (*Mus musculus*) and some strains of rats (*Rattus norvegicus*), show impressive elaboration of a specific tissue of the submandibular gland, the granulated convoluted tubular (GCT) tissue, often only in males following puberty [21]. This sex-limited tissue differentiation causes the submandibular glands with elaborated GCT to produce kallikrein serine proteases encoded in *Klk1* gene subfamilies that have recently expanded independently in house mice and rats [14]. This results in a clear sex-limited expression of all, but a few, of these *Klk1b* subfamily kallikrein genes in male mice, but the picture is not so clear in rats [2]. The two strains of rat that have been studied to date show a very different expression of their *Klk1c* subfamily kallikrein genes, with the genome rat not expressing any of them, while the Sprague-Dawley rat expresses the *Klk1c* kallikrein genes in both sexes. Unfortunately, neither human saliva proteome project [3,4] addressed the issue of differential expression of proteins in males and females. Thus, we cannot currently assess the contribution of sex-limited expression to the complement of proteins found by [4] that were not found by [3,4].

### 3.5. Proteins Unique to Each Saliva Proteome

Removing the salivary proteins shared by two or three of the mammal proteomes allowed the identification of the proteins unique to each of them (SF4, SF5). The human saliva proteome contains a number of salivary proteins that distinguish it from the rodent proteomes, including the statherin-like PRPs, the histatins, zinc alpha glycoprotein and the Ig saliva secretory complex. Statherin prevents calcium phosphate precipitation in saliva, thus allowing calcium to be maintained at a supersaturated level in saliva to prevent deterioration of the teeth [22]. In addition to the physical shielding properties of the epithelial layer and mucin, components of innate immunity including lysozyme, lactoferrin and cystatins likely cooperate with adaptive humoral immunity mediated by antibodies in the Ig secretory complex to fight infection in the human oral cavity [23]. The presence of lysozyme and the Ig secretory complex in human, but not in rodent, saliva suggests that humans have more need of such weapons against infection. The remaining proteins appear to have an assortment of unrelated functions. Strikingly, the addition of the proteins unique to [3] and to [4] that have signal peptides brought the human list to 381. A brief survey of these proteins produced descriptions, such as: uncharacterized protein, protein existence uncertain and tissue specificity = epidermis, protein existence inferred from homology, and subcellular location = lysosome. In other words, the majority of these protein identifications seem to make up a highly heterogeneous collection of proteins, and we suspect that many of them are contaminants in spite of having signal peptides.

Of the 22 unique mouse salivary proteins, 2/3 consist of eleven *Klk1b*-encoded subfamily kallikreins and three androgen-binding protein (ABP) subunits (total of 14), none of which have human equivalents. The *Klk1b* subfamily kallikreins are expressed almost exclusively in males, and we have suggested, on the basis of new data, that the previous speculative function of the species-specific rodent kallikreins as important solely in wound healing in males be investigated further. In addition to or instead of that function, we proposed that their sex-limited expression, coupled with their rapid evolution, may be clues to an as-yet-undetermined interaction between the sexes [2]. The three ABP subunit proteins, which form dimers to produce mouse pheromones (reviewed in [24]), are found in both sexes of mice and have been proposed to be involved in incipient reinforcement, where subspecies of mice make secondary contact [17]. Mice also secrete trypsinogen, a peptidase inhibitor, MUP5, EGF binding protein, vomeromodulin, a glycoprotein and two poorly characterized proteins.

The genome rat saliva proteome has only three unique proteins: contiguous repeat polypeptide, an alpha-2 microglobulin distinct from the shared version, and an uncharacterized protein with similarity to GRPCB. Although, as we noted above, the saliva of another rat strain also contains numerous rat-specific kallikreins. Thus, the question of whether the expression of species-specific kallikrein family genes is shared between the two rodents or unique to mice depends on the strain of rat in the comparison. 

## 4. Conclusions

Much work has been done on individual salivary proteins in humans and other animals over the past five decades, and there are relatively recent research papers and reviews that have focused on the human salivary proteins (e.g., PRPs [25]; a human saliva glycoprotein proteome [26]; a human proteomic study from a consortium of institutions [4]; and a 2013 review of human salivary proteins [27]). Less has been done with rodent salivary proteins. We published the results of the application of multidimensional protein identification technology (MUDPIT), an LC-LC-MS/MS analysis, to stimulated mouse and rat saliva for the purpose of studying rapidly evolving proteins and their genes [2]. 

It is possible that the comparison and contrast of the salivary protein components of human and rodent saliva that we have presented here has raised more questions than it has provided insights. Given that there has been no previous such study, we hope that at least we have framed some important questions, especially evolutionary ones, for us and for others to pursue. Our one conclusion that we feel will be useful for future studies involving one or the other rodent as a model for human oral physiology is that there are significant differences in the protein constituents between the salivas of humans and rodents, which could be misleading if not taken into consideration. 

## Supplementary Materials

Supplementary File 1

## Supplementary Files

SF1: Human salivary proteins shared by [3,4] with SignalP and shared expression status;
SF2: Human salivary proteins unique to [4] with signal peptides, listing shared expression;
SF3: Human salivary proteins unique to [3] with SignalP and shared expression status;
SF4: Mouse salivary proteins [2] with SignalP and shared expression status;
SF5: Rat salivary proteins [2] with SignalP and shared expression status.
